# Quality and psychosocial factors influencing purchase of orange‐fleshed sweetpotato bread

**DOI:** 10.1111/ijfs.14822

**Published:** 2020-11-05

**Authors:** Julius Juma Okello, Felix Mwala Shiundu, Janet Mwende, Carl Johan Lagerkvist, Rose Adhiambo Nyikal, Penina Muoki, John Mburu, Jan Low, Guy Hareau, Simon Heck

**Affiliations:** ^1^ International Potato Center P.O. Box 106 Kampala Uganda; ^2^ Department of Agricultural Economics University of Nairobi P.O. Box 29053‐00625 Kangemi Nairobi Kenya; ^3^ International Potato Center Kisumu Liaison Office P.O. Box 1745‐40100 Kisumu Kenya; ^4^ Department of Economics Swedish University of Agricultural Sciences P.O. Box 7013 Uppsala 75007 Sweden; ^5^ International Potato Center P.O. Box 25171 Nairobi Kenya; ^6^ International Potato Center Av. La Molina 1895, La Molina Lima 12 Peru

**Keywords:** Consumer acceptance, orange‐fleshed sweet potato bread, psychosocial drivers, quality attributes, urban consumers

## Abstract

This 2018 study, conducted in six Tusky's supermarkets in Nairobi, Kenya, combined the Just‐About‐Right, Penalty and Mean‐End‐Chain analyses to examine the quality and psychosocial factors influencing the purchase of a novel bread made from orange‐fleshed sweet potato (OFSP), a biofortified crop, focusing on sixty‐one male and eighty female urban OFSP bread buyers recruited at point of purchase. It finds that sensory and psychosocial factors drive purchasing decisions and that some of the bread's sensory characteristics are misaligned with consumers' expectations. It also finds that women and men’s evaluations of the bread's characteristics are different, as are their motivations for purchase. However, good sensory attributes and the knowledge of the bread's nutritional value were key drivers. Some misaligned characteristics reveal levers for the reformulation of the bread and present opportunities for segmenting the market. Several other implications of the findings for policy and future improvement of the bread are discussed.

## Graphical abstract

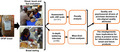

## Introduction

Biofortified foods are being promoted in many African and Asian countries as a low cost, effective option for tackling micronutrient deficiency among rural households (Low & Thiele, 2020). The main vehicle for delivering the essential micronutrients has been fresh produce grown and consumed by farm households, with surplus sold. In Africa, the biofortified crops are orange‐fleshed sweet potato^1^ (OFSP; for vitamin A), high‐iron beans (for iron), yellow cassava (vitamin A) and pearl millet (for iron). Households receive planting material ideally combined with nutrition and agronomic information packaged to encourage cultivation and consumption of these nutrient‐dense crops.

More recently in Africa, efforts have expanded into developing value chains for industrially processed foods as an additional vehicle for delivering biofortified foods, specifically to non‐farm households, in recognition that the African continent is the fastest urbanising continent (Low *et al*., 2017b) and to enhance market opportunities for farmers. A case in point is the OFSP bread, first launched in Kenya in 2015 by a private‐sector bakery and leading supermarket chain, Tuskys, targeting mainly urban consumers in Nairobi, the capital city, as part of facilitated private–public partnership led by the International Potato Center (Bocher *et al*., 2017). Since then, OFSP bread has been launched in urban cities of Ghana, Malawi, Mozambique and Ethiopia. Little is known about what drives or influences urban consumers to purchase and consume them and the characteristics of those consumer segments that prefer OFSP bread to the standard 100% wheat flour bread. Current evidence about the preference for biofortified foods is limited in scope with a majority of past studies relying on hypothetical choice studies (Naico & Lusk, 2010; Chowdhury *et al*., 2011); or real taste tests of fresh products presented to consumers at their homes (Tomlins *et al*., 2007; Lagerkvist *et al*., 2016), in a school setting (Laurie *et al*., 2018) or at grocery stores (de Groote *et al*., 2014). The exceptions are Ouro‐Gbeleou (2018), Bocher *et al*. (2019) and Wanjuu *et al*. (2019), who used econometric techniques and neoclassical economic theories to assess drivers of OFSP acceptance, focusing on only a small range of sensory attributes. While such analyses provided useful evidence, consumer studies have pointed to a need to extend the analysis of consumer choices and preferences beyond the rational behavioural assumptions of the neoclassical economic analysis (Grunert, 2019; Jaeger *et al*., 2019). Low *et al*. (2015) explored the state of knowledge concerning consumer behaviour and food choice, calling on learning from the private‐sector marketing firms on how they examine urban consumer food choice by studying personal, psychological, social, economic and environmental drivers. More widely used marketing techniques could be drawn on to understand better consumers' assessment and acceptance of products, especially those in the early stages of launch (Banović *et al*., 2016; Rocha *et al*., 2019).

In this paper, we employed consumer marketing tools and principles of economic psychology to analyse quality and psychosocial factors influencing the acceptance and decision to purchase OFSP bread. First, we explored what quality attributes influence choice decisions. Second, we evaluated the importance of each of the quality attributes identified in the decision to purchase OFSP bread. Third, employing the principles of economic psychology, we investigated the life goals or personal motivational aspects that drive the purchase of OFSP bread instead of the regular wheat bread. Thus, the study combined quantitative and qualitative analysis techniques to systematically assess the drivers of the decision to purchase OFSP bread. Given that the product is still relatively new in many markets, the goal is to provide valuable information for improving the recipes for OFSP bread and other OFSP‐based commercial products and the design of their promotional messages to increase demand. This study, therefore, contributes to better understanding of the sensory attributes most desired by urban bread consumers, providing pertinent information for the refinement and promotion of OFSP bread.

### Study background

The development and commercialisation of the OFSP bread in Kenya was part of the strategy for diversifying the use of orange‐fleshed sweet potato roots. Biofortified sweet potato varieties were introduced in Kenya in 1995. Starting in 2009, Kenya was part of a seventeen country initiative in sub‐Saharan Africa, to distribute high‐quality planting material of improved varieties to combat malnutrition and poverty (Low, 2011). Vitamin A deficiency (VAD) is especially prevalent among young children under 5 years of age, pregnant women and breastfeeding mothers (Low *et al*., 2017a). Vitamin A is an essential micronutrient for assuring a strong immune system and healthy eyesight, and an indicator of VAD is when serum retinol levels fall below 0.70 µmol L^−1^ (Sommer & West, 1996). Several studies have confirmed the efficacy of OFSP in combating VAD among children under five years of age and fighting diarrhoea when combined with a community level nutrition education campaign (Low *et al*., 2007; Hotz *et al*., 2012; Jones & de Brauw, 2015; de Brauw *et al*., 2018).

Research done in Mozambique demonstrated that OFSP bread, having 38% of wheat flour replaced (by weight) by OFSP purée (steamed and mashed sweet potato), met the criteria of being a good source of vitamin A^2^ when at least medium‐intensity orange varieties were used (Low & van Jaarsveld, 2008). In 2014, a scaling up project led by the International Potato Center (CIP) – Scaling Up Sweet potato for Agriculture and Nutrition (SUSTAIN) – embarked on commercialising the OFSP value chain in Kenya targeting non‐farm households with value‐added products.

To support ease of use of OFSP as an ingredient, an OFSP purée processing company – Organi Ltd (Kabondo, Homa Bay, Kenya) – was established in western Kenya to process fresh roots into a versatile packaged product that facilitates ease of use by bakeries (Okello, 2019). At the same time, a public–private partnership initiative involving a national supermarket chain, Tuskys, a Kenyan private investor (Organi Ltd), a food technology firm (Euro‐Ingredients Ltd), and an international research organisation (CIP), embarked on the development of OFSP purée based bakery products, culminating in the launch of OFSP bread, buns and Gallet bread in 2015, on a pilot basis (Bocher *et al*., 2017). The bread was baked in Tuskys main/central bakery based in Nairobi and sold in Nairobi outlets only, with the aim of expanding to other outlets outside Nairobi. The bread replaced 43% of wheat flour (by weight) with OFSP purée (Table 1). The variety used for the purée is Kabode. The bread sold for five Kenyan shillings (KES) more than the 100% wheat flour bread, as Tusky's viewed it as a niche product for health‐conscious consumers. That is, at the time of study the retail price of OFSP bread was KES 50 (0.50 USD)^3^ for the 400 g and it was sold in a clear plastic packaging. In comparison, the standard 400 g wheat bread was selling at a price of KES 45 (0.44 USD) and was also in clear branded packaging. Studies have shown that two slices of such bread could provide 30% of the daily recommended dietary allowance of vitamin A for children below 3 years of age (Muzhingi *et al*., 2016; Nzamwita *et al*., 2017; Awuni *et al*., 2018). Using an OFSP purée as a functional ingredient in bread and other bakery products for the urban market added value to these frequently consumed products and widened access to the nutritional benefits of OFSP beyond OFSP root consumption (Fonzi & Nouwen, 2018). In East Africa, sweet potato traditionally has been viewed as a crop of the poor; however, in cities, more health‐conscious consumers recognised the value of nutrient‐rich foods such as sweet potato and demand has been increasing (Low *et al*., 2017b).

**Table 1:** Ingredients for 90 loaf (400 g each) batch of orange‐fleshed sweet potato (OFSP) bread* and standard white bread recipes

Ingredient	OFSP bread	Standard white bread
	Quantity (g)	Quantity (g)
Wheat flour	23 000	40 250
OFSP purée	17 250	
Sugar	430	1800
Salt	430	450
Yeast (brand: mauripan)	450	280
Bread improver	200	100
Fat (brand: veebol vegetable oil)	1200	1800
Functional gluten	300	
Water	5 L	10 L

* Procedure for preparation: (i) add all ingredients into the mixer except the water; (ii) mix everything for 15 min, adding water slowly while testing dough strength; (iii) remove dough, knead, and cut dough into pieces of 450 g each; (iv) oil loaf mould, and put cut pieces in moulds; (v) load moulds into proofer for 30 min; (vi) Bake at 191–271 °C for about 30 min; (vii) remove loaves from moulds and cook 15 min before slicing and packing.

At the time of this study, this commercial OFSP bread value chain was still in its infancy, with sales concentrated in Tuskys Supermarket outlets in Nairobi. Baking of the bread was centrally controlled and sometimes intermittent due to management issues. The familiarity among consumers in Kenya with OFSP and its nutritional benefits was still limited, with roots of the white and yellow‐fleshed varieties containing little to no vitamin A, still dominating the consumption landscape owing to their popular firm texture (high dry matter) and taste.

Previous studies on acceptance of OFSP‐based products (Bocher *et al*., 2017; Ouro‐Gbeleou, 2018; Bocher *et al*., 2019; Wanjuu *et al*., 2019) found that certain OFSP varieties have a different flavour to the white and yellow‐fleshed counterparts, and have documented differences over the sensory spectrum especially relating to smell, appearance, taste and texture (Tomlins *et al*., 2012). Stevens & Winter‐Nelson (2008) also found that the distinct orange colour of OFSP, due to its high content of beta‐carotene, evokes expectations of an inferior taste of boiled roots in Mozambique. Muzhingi *et al*. (2018) argue that the addition of OFSP purée resulted in a bread with a desirable golden and thicker crust, which he attributed to higher moisture content in the bread. Our study evaluated urban consumers' assessment of a wide range of bread attributes/characteristics and investigated the role psychosocial factors play in the decision‐making process. It also examined differences in OFSP bread evaluation and purchase decision process by urban male and female consumers. A brief review of literature revealed that few bakery product evaluations have looked at gender differences, yet in some developing countries such as Kenya, men most often control the use of family resources (including money) while women are also involved in food purchase (Crane *et al*., 2019; Ragasa *et al*., 2019).

## Materials and methods

This study used three analytical approaches, namely the Just‐About‐Right (JAR) scale, the Penalty Analysis (PA) and Mean‐End‐Chain analysis. Each is described briefly below.

### Analytical approach – the JAR and PA

The JAR scale captures consumer assessment of the appropriateness or adequacy of a range of quality attributes during product optimisation (García‐Díez *et al*., 2017). The JAR scale is widely used by marketers in the early stages of the product development to gauge the optimum level of attributes to offer (Lawless & Heymann, 1998; Luc *et al*., 2020). The scale is also employed to improve the design of a product already in the market to expand its market share.

In this study, the appropriateness of seventeen sensory characteristics of the OFSP bread was scored on a 5‐item bipolar hedonic JAR scale, used by Rothman & Parker (2009), ranging from ‘too little’ (−2) to ‘too much’ (+2) with the JAR (0) as the ideal‐level centre‐point. Frequencies for each scale were estimated using Stata software, with 70% as the recommended threshold for acceptability (Stone & Sidel, 2004) of each sensory characteristic.

The PA is typically used in association with the JAR scales by practitioners/product designers to identify decreases in acceptability associated with sensory attributes, not at optimal levels in a product (Ares *et al*., 2014). The PA also requires the collection of ‘liking’ data captured using a 7 or 9‐point hedonic scale. We assessed the actual liking of the OFSP bread in this study on a 7‐point hedonic scale ranging from ‘dislike very much’ (1) to ‘like it very much’ (7).

Using the PA, we identified a prioritised list of critical product attributes that most penalise OFSP bread, thus decreasing demand. We present the penalties or ‘mean drops’ to the OFSP bread as reduced overall liking for not being ‘just‐about‐right’ on a specific attribute. First, we divided the respondents into three groups depending on the response to a JAR attribute (i.e. Too little, JAR or Too much). Then, we calculated the percentage of consumers in each group and estimated the corresponding mean liking scores for the ‘Too Little’ (TL), ‘JAR’ and ‘Too Much’ (TM) categories as follows: Penalty (mean drops) for TL (Penalty TL) = JAR − TL and Penalty for TM (Penalty TM) = JAR − TM. Finally, we visualised the results of the analysis highlighting the characteristics that were most essential to increase overall OFSP bread acceptability.

### Means‐end chain analysis

The means‐end chain (MEC) analysis draws from economic psychology (Gutman, 1982; Reynolds & Olson, 2001) to assess consumer motivation to choose/consume a product (Santosa & Guinard, 2011). It has been widely deployed during the early stages of product development and launches to examine design aspects or quality characteristics influencing consumer choice decisions.

Means‐end chain analysis posits that product characteristics (usually known as *attributes*) are linked to perceived benefits (also called *consequences*) in the sub‐conscious minds of consumers. These *consequences* are mentally associated with personal life goals (known as *values*) that consumers aspire to fulfil in life. *Attributes* are the recognisable characteristics (e.g. taste and texture) of a product (Okello *et al*., 2014). *Consequences* are the desired outcomes (benefits) an individual obtains from consuming a given product (Arsil *et al*., 2014). *Values* represent the life goals or desires that motivate a product choice (Mason, 1995; Lind, 2007). Aarts *et al*. (2008) and Lagerkvist *et al*. (2020) argue that goals can stimulate or activate action/decision. The *attribute*‐*consequence*‐*value* (A‐C‐V) links form the MEC hierarchies or Hierarchical Value Map (HVM). HVM is a mental representation of how the product's features, perceived benefits and motivating life goals are organised in a consumer's mind.

In this study, we generated the *attributes*, *consequences* and *values* that formed HVMs using an interview process known as laddering (Reynolds & Gutman, 1988; Russell *et al*., 2004; Pambo *et al*., 2017). The interview commenced with the following statement:

We are interested in what comes to your mind when considering the following question (please note that the results will be anonymous): What attributes/features/characteristics made you choose to buy and consume the sweetpotato (i.e. OFSP) bread?

After identifying the (quality) characteristics (i.e. *attributes*) motivating the decision to buy the OFSP bread, we asked the respondent to ‘Rank the attributes/features/characteristics you have listed in the order in which you consider them when purchasing the sweetpotato bread’. We then used the top three ranked *attributes* to construct the laddering interview, starting with the highest‐ranked. We used a series of ‘Why is that important to you?’ questions, occasionally followed by probes, to extract the mental constructs behind the purchasing decision. Use of probes aided in ensuring the revelation of all the mental linkages/associations and completion of the ladders (i.e. the A‐C‐V links).

Finally, we used content analysis to note the *attributes*, *consequences* and *values* obtained, and develop summary codes, grouping together and merging closely related constructs to enhance the efficient interpretation of the total number of constructs. The summary codes were then entered and analysed using the MECAnalyst software. The software produced HVM values discussed in this study, for the full aggregated sample and by gender of the OFSP bread purchaser.

### Sensory attributes

The sensory characteristics assessed were sweetness/sugariness, sweet potato smell, the smell of bread (aroma), yellow colour, wheat flavour, saltiness, soft texture, airy texture, smooth texture, golden crust, sweet potato flavour, hard texture, slice heaviness, compactness, shape, sourness/sourdough and mouthfeel (focused on stickiness when chewing). These characteristics were obtained from findings from two pre‐survey focus group discussions (FGDs) involving twenty bread consumers, held at the University of Nairobi in April 2018. The FGDs also identified some interesting sensory observations: (i) a sweet potato smell as well as sourness was associated with bread being stale or expired/bad; (ii) brown/whole grain bread was associated with non‐acceptable texture; (iii) golden brown crust was associated with well‐baked bread.

### Sampling and data

The study was conducted during May 2018 in Tuskys supermarket stores in Nairobi, Kenya. Tuskys only sold OFSP bread in Nairobi stores. Six Tuskys supermarket stores were purposively selected in areas of the city where low, middle and high‐income consumers are likely to shop (in case of those in the central business district) or reside (for those in or near residential areas), namely Hakati and Chap representing low‐income customers, Buruburu and Pioneer with predominantly middle‐income customers, and T‐Mall and Karasha with higher‐income customers.

The respondents were recruited in‐store at the point of purchase using systematic random sampling. That is, every second buyer of OFSP bread was selected for interviews in each store until the quota for the day of at least six respondents was attained. The daily quota was set at six because every store had two enumerators each interviewing three respondents a day. Recruitment at point of purchase minimises the problem of reluctance or lack of confidence to provide true feelings and opinions during food evaluation tasks reported for African consumers (Ramoroson Rakotosamimanana & De Kock, 2020). The in‐store environment is also likely to induce behaviour aligned to the shopper's self‐interest because it is a familiar shopping environment, thus contributing to improved external validity (List, 2003). It is the natural environmental context in which real shopping behaviour can be directly observed (Carrol & Samek, 2018). Interviews were conducted at the bread section in each store. A total of 141 OFSP bread purchasers (sixty‐one male; eighty female) were interviewed across the six stores.

Laddering interviews were conducted on a sub‐sample of fifty‐five of the respondents selected randomly at each store. Each day, a trained research assistant randomly selected 2–3 respondents for additional in‐depth laddering interview at one of the stores and rotated around all the six stores. Every second buyer of the OFSP bread that completed the first interview was selected for laddering interviews. However, four of the respondents did not fully complete the laddering interviews and were excluded from the analysis. Although this sample size was small, it compares well with previous MEC studies reported by Pambo *et al*. (2017) (*n* = 54), Okello *et al*. (2014) (*n* = 56) and Schaefers (2013) (*n* = 14).

Interviews were conducted from 8 am to 7 pm during weekdays and on weekends to capture different shopping patterns. The interview team comprised eleven enumerators (six females, five males) supervised by two Research Assistants (one female, one male). Prior to the interview, informed written consent was obtained from each respondent. The male Research Assistant conducted the laddering interviews.

### Visual observation and tasting of OFSP bread

Each respondent, during the interview process, was allowed to assess visually – via touch and feel – the loaf colour, firmness, crust colour and to taste the OFSP bread. Before tasting the OFSP bread, respondents were offered a cup of still water at room temperature to rinse their mouths. The bread used in the study was obtained from the store shelf on each study day and confirmed to be within the ‘use‐by’ date before use.

## Results and discussion

### Characteristics of the respondents

Summary statistics (Table 2) indicate that on average, both male (34.7 years) and female (36.4 years) respondents were middle‐aged, had more than 14 years of formal schooling (post‐secondary education) and fell within the low‐income category. As expected, more males (78%) were household heads^4^ than females (23%). Almost two‐thirds of the male respondents were salaried employees, compared to almost half of the female respondents. This implied that more male respondents in the sample had regular income sources to spend on food than female counterparts. Being ahead of household and/or having salaried income no doubt has a strong influence on individual’s purchasing power, hence food purchase and consumption decisions.

**Table 2:** Sociodemographic characteristics of respondents in bread study

	Overall (*N* = 141)	Male (*N* = 61)	Female (*N* = 80)
	Mean	Mean	Mean
Age (years)	35.2 (13.1)	34.7 (12.01)	36.4 (12.72)
Education (years)	14.8 (2.5)	15.1 (2.52)	14.5 (2.45)
Relation to household head (%)			
Self	53	78	23
Spouse	40	15	58
Son/daughter	6	5	6
Brother/sister	2	1	1
Niece/nephew	0	2	1
Occupation (%)			
Salaried (full time)	55	65	47
Part time	11	7	15
Trader/business	16	8	21
Retired	2	3	1
Student	9	10	7
Jobseeker	7	7	7
Housewife	1	0	1
Marital status (%)			
Single	40	40	40
Married	58	60	57
Separated	1.4	0	2
Widow/widower	0.7	0	1
Income level (%)			
<76 500 KES* per month (low)	61.7	55	67
76 500–102 500 KES per month (middle)	29.1	28	30
>102 500 KES per month (high)	9.2	17	4

* At the time of study USD 1 ≈ Kenya Shillings (KES)100; numbers in parentheses are standard deviations.

Table 3 presents summary statistics for the laddering interview respondents. Slightly more females (61%) participated in the laddering interviews than males (39%). As in the full sample, the respondents were, on average, middle‐aged and had a post‐secondary level of education. Slightly more than one‐half (55%) were on salaried employment. However, more female respondents (87.10%) fell in the low‐income category as compared to male respondents (50%). On average, the laddering respondents had four household members, with only 19.61% and 1.96% of the households having a breastfeeding or pregnant member, respectively. About one‐fifth of respondents were in households with two often targeted groups for risk of VAD: children less than 2 years and breastfeeding women. The number of respondent households with the other major vulnerable group, pregnant women, was small (2% of total). Having members who are vulnerable to VAD in the household could potentially positively influence the decision to purchase OFSP bread.

**Table 3:** Descriptive statistics of laddering interviews of OFSP bread purchasers

Variable	Overall (*n* = 51)	Female (*N* = 31)	Male (*N* = 20)
	Mean	Mean	Mean
Age of respondent (years)	38.5 (12.93)	37.1 (13.39)	40.7 (12.20)
Household size (count)	4.3 (2.16)	4.3 (2.45)	4.3 (1.63)
Education (years)	15.1 (2.25)	15.0 (2.11)	15.4 (2.50)
Occupation (salaried) (%)	54.9	51.6	60.0
Income (low category) (%)	72.6	87.1	50.0
Pregnant member in household (%)	2.0	3.2	0.0
Marital status (married) (%)	66.7	58.1	80.0
Relation with household head (self) (%)	62.8	38.7	100.0
Child below 6 months of age present in household (%)	2.0	3.2	00.0
Child below 2 years of age present in household (%)	21.6	12.9	35.0
Child below 5 years of age present in household (%)	35.3	78.0	72.5
Breastfeeding member present in household (%)	19.6	16.1	25.0

* Numbers in parentheses are standard deviations.

### Quality attributes influencing the purchase of OFSP bread

Table 4 has captured the respondents' assessment of the appropriateness of the various OFSP bread characteristics by gender, together with the Mann–Whitney test results for significant differences between genders. For both genders, sweet potato smell, sweet potato flavour, hard texture and sourness are the characteristics with the most substantial deviation from the 70% recommended threshold for acceptability (Stone & Sidel, 2004). The respondents assessed these characteristics of the OFSP bread as being ‘too little’.

**Table 4:** Per cent of OFSP bread purchasers in different Just‐about‐Right (JAR) categories by sensory characteristic and gender

	Per cent of JAR counts					Mann–Whitney test (*P*‐value) Female vs. male
	Much too little	Too little	Just‐about‐right	Too much	Much too much	
Female (*N* = 80)						
Sweetness	1.2	13.6	79.0	6.2	0.0	0.452
Sweet potato smell	19.8	39.5	**35.8**	3.7	1.2	0.380
Bread smell	4.9	8.6	81.5	4.9	0.0	0.680
Yellow colour	1.2	7.4	80.2	8.6	2.5	0.411
Wheat flavour	2.5	24.7	**63.0**	7.4	2.5	0.296
Saltiness	9.9	8.6	**67.9**	11.1	2.5	0.712
Soft texture	0.0	1.2	75.3	16.0	7.4	0.920
Airy texture	2.5	17.3	**65.4**	11.1	3.7	0.634
Smooth texture	0.0	3.7	79.0	11.1	6.2	0.988
Golden crust	1.2	6.2	77.8	12.3	2.5	0.285
Sweet potato flavour	18.5	35.8	**39.5**	6.2	0.0	0.846
Hard texture	30.9	25.9	**42.0**	0.0	1.2	0.380
Slice heaviness	2.5	8.6	82.7	4.9	1.2	**0.005**
Compactness	6.2	3.7	86.4	3.7	0.0	**0.099**
Shape	0.0	7.4	91.4	0.0	1.2	0.286
Size weight	4.9	9.9	79.0	3.7	2.5	0.438
Sourness	11.1	21.0	**56.8**	8.6	2.5	0.748
Mouthfeel	6.2	4.9	72.8	13.6	2.5	**0.040**
Male (*N* = 61)						
Sweetness	11.7	3.3	80.0	5.0	0.0	
Sweet potato smell	16.7	35.0	**41.7**	6.7	0.0	
Bread smell	6.7	15.0	**68.3**	8.3	1.7	
Yellow colour	0.0	8.3	86.7	5.0	0.0	
Wheat flavour	8.3	21.7	**66.7**	1.7	1.7	
Saltiness	6.7	8.3	71.7	11.7	1.7	
Soft texture	1.7	1.7	71.7	13.3	11.7	
Airy texture	3.3	18.3	66.7	6.7	5.0	
Smooth texture	0.0	3.3	80.0	8.3	8.3	
Golden crust	0.0	13.3	75.0	10.0	1.7	
Sweet potato flavour	18.3	35.0	**38.3**	8.3	0.0	
Hard texture	25.0	25.0	**48.3**	1.7	0.0	
Slice heaviness	8.3	20.0	70.0	1.7	0.0	
Compactness	3.3	16.7	78.3	1.7	0.0	
Shape	1.7	10.0	88.3	0.0	0.0	
Size weight	3.3	11.7	85.0	0.0	0.0	
Sourness	15.0	13.3	**58.3**	13.3	0.0	
Mouthfeel	6.7	1.7	**60.0**	21.7	10.0	

Bolded values indicate key distinctions discussed further in the text.

Both categories of respondents also found wheat flavour in the OFSP bread to be ‘too much’ (Table 4). This finding, when combined with the above about sweet potato flavour, suggested that the respondents having learned that the bread they were evaluating was made from OFSP, expected to feel/sense a stronger sweet potato flavour in the bread.

Results further indicated that both male and female purchasers found the OFSP to be rather too soft and rated soft texture as ‘too much’. Combining this finding with the earlier one relating to hard texture indicated that there is room to adjust the proportions of wheat flour and OFSP purée in the bread. An earlier finding by Wanjuu *et al*. (2018) indicated that a higher proportion of OFSP purée increases the smoothness of the crumbs, suggesting that the proportions can be adjusted so that more OFSP purée is used than the current 43%. Thus, increasing the proportion of OFSP purée in the bread would have both functional, nutritional and economic benefits, the latter arising from foreign exchange savings as Kenya is a net wheat importer.

Many respondents also felt mouthfeel to be excessive (‘too much’), with male respondents showing more variation in this trait than their female counterparts. Indeed, in addition to compactness and slice heaviness, this was the trait for which there was a clear distinction in acceptability rating between female and male respondents, as shown by the Mann–Whitney test (*P*‐value < 10%; last column of Table 4). In addition, only about 8.6% of female respondents found slice heaviness to be ‘too little’ as compared to a much larger percentage (20%) of their male counterparts. In the case of compactness, there were clear gender differences in the proportions of purchasers finding the bread to have ‘too little’ compactness (female = 3.7%; male = 16.7%) and those finding compactness to be ‘much too little’ (female = 6.2%; male = 3.3%).

**Figure 1:**
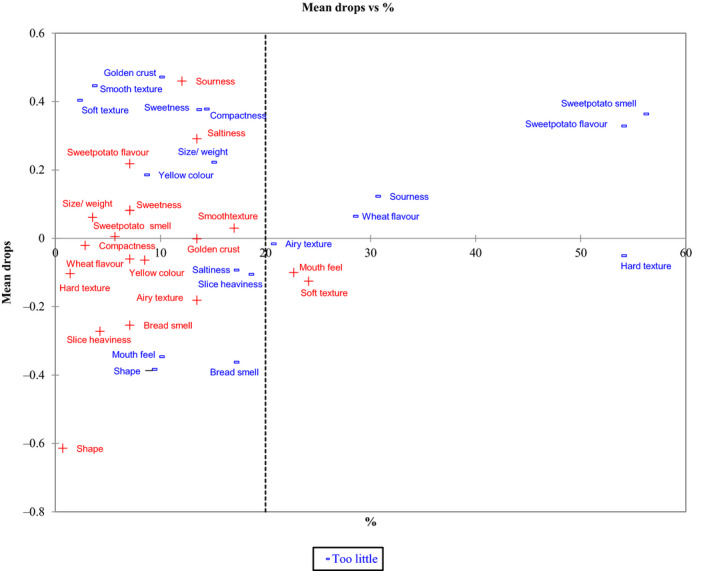
Consumers’ evaluation of orange‐fleshed sweet potato bread characteristics using penalty analysis (*N* = 141).

We estimated the means drops for the levels that are not JAR (i.e. ‘too much’ and ‘too little’) penalties are shown in Table 5. Mean drops represent the difference between the mean overall liking for the JAR levels and the ‘too much’ or ‘too little’ levels. Figure 1 summarises the results of the penalty analysis. The plot is divided into four subplots using a vertical line representing 20% of the purchasers. The horizontal axis presents the percentage of respondents rating the product characteristic, while the mean drops are presented in the vertical axis. The upper right subspace contains the important attributes (with more than 20% of respondents' ratings), which need to be emphasised during the product reformulation or refinement of the bread. The figure shows that nearly 60% of the respondents found sweet potato smell, sweet potato flavour and hard texture to be ‘too little’. On the other hand, more than 20% of the respondents found the mouthfeel and soft texture ‘too much’.

**Table 5:** Penalty table of sensory characteristics of OFSP bread evaluated by purchasers

Characteristic	Level	Frequencies	%	Sum (liking)	Mean (liking)	Mean drops	Penalties
Sweetness	Too little	19	13.48	116.000	6.105	0.377	
	JAR	112	79.43	726.000	6.482		0.275
	Too much	10	7.09	64.000	6.400	0.082	
Sweet potato smell	Too little	79	56.03	495.000	6.266	0.364	
	JAR	54	38.30	358.000	6.630		0.331
	Too much	8	5.67	53.000	6.625	0.005	
Bread smell	Too little	24	17.02	161.000	6.708	−0.363	
	JAR	107	75.89	679.000	6.346		−0.331
	Too much	10	7.09	66.000	6.600	−0.254	
Yellow colour	Too little	12	8.51	75.000	6.250	0.186	
	JAR	117	82.98	753.000	6.436		0.061
	Too much	12	8.51	78.000	6.500	−0.064	
Wheat flavour	Too little	40	28.37	255.000	6.375	0.065	
	JAR	91	64.54	586.000	6.440		0.040
	Too much	10	7.09	65.000	6.500	−0.060	
Saltiness	Too little	24	17.02	157.000	6.542	−0.093	
	JAR	98	69.50	632.000	6.449		0.077
	Too much	19	13.48	117.000	6.158	0.291	
Soft texture	Too little	3	2.13	18.000	6.000	0.404	
	JAR	104	73.76	666.000	6.404		−0.083
	Too much	34	24.11	222.000	6.529	−0.126	
Airy texture	Too little	29	20.57	186.000	6.414	−0.016	
	JAR	93	65.96	595.000	6.398		−0.081
	Too much	19	13.48	125.000	6.579	−0.181	
Smooth texture	Too little	5	3.55	30.000	6.000	0.446	
	JAR	112	79.43	722.000	6.446		0.102
	Too much	24	17.02	154.000	6.417	0.030	
Golden crust	Too little	14	9.93	84.000	6.000	0.472	
	JAR	108	76.60	699.000	6.472		0.199
	Too much	19	13.48	123.000	6.474	−0.001	
Sweet potato flavour	Too little	76	53.90	478.000	6.289	0.329	
	JAR	55	39.01	364.000	6.618		0.316
	Too much	10	7.09	64.000	6.400	0.218	
Hard texture	Too little	76	53.90	490.000	6.447	−0.051	
	JAR	63	44.68	403.000	6.397		−0.052
	Too much	2	1.42	13.000	6.500	−0.103	
Slice heaviness	Too little	26	18.44	169.000	6.500	−0.106	
	JAR	109	77.30	697.000	6.394		−0.137
	Too much	6	4.26	40.000	6.667	−0.272	
Compactness	Too little	20	14.18	122.000	6.100	0.379	
	JAR	117	82.98	758.000	6.479		0.312
	Too much	4	2.84	26.000	6.500	−0.021	
Shape	Too little	13	9.22	88.000	6.769	−0.383	
	JAR	127	90.07	811.000	6.386		−0.400
	Too much	1	0.71	7.000	7.000	−0.614	
Size weight	Too little	21	14.89	131.000	6.238	0.223	
	JAR	115	81.56	743.000	6.461		0.192
	Too much	5	3.55	32.000	6.400	0.061	
Sourness	Too little	43	30.50	275.000	6.395	0.123	
	JAR	81	57.45	528.000	6.519		0.219
	Too much	17	12.06	103.000	6.059	0.460	
Mouthfeel	Too little	14	9.93	94.000	6.714	−0.346	
	JAR	95	67.38	605.000	6.368		−0.175
	Too much	32	22.70	207.000	6.469	−0.100	

Another OFSP bread characteristic than could be tweaked is ‘size weight’ which was rated as ‘too little’ by slightly more than 15% of the respondents. Participants argued that larger families preferred buying a single bigger ‘family‐size’ loaf of bread enough to feed everybody than buying two leaves which could cost more. At the time of this study, Tuskys produced and sold only 500 g size loaves, which are much smaller than the one kg ‘family‐size’ loaf. Thus, this finding suggests that future OFSP bread refinement should diversify production to include the ‘family‐size’ loaf option. In addition, sweetness and saltiness were both rated as ‘too little’ by more than 15% of the respondents. This assessment of the OFSP bread indicated an emerging opportunity for its marketing as a niche product that targets the sub‐population of bread consumers that are health‐conscious/seeking as relates to sugar and salt intakes, including those likely to be diagnosed with or are concerned about diabetes and hypertension. These characteristics, therefore, provided OFSP bread manufacturers a tool for market segmentation.

### Psychosocial drivers of the decision to purchase OFSP bread

**Figure 2:**
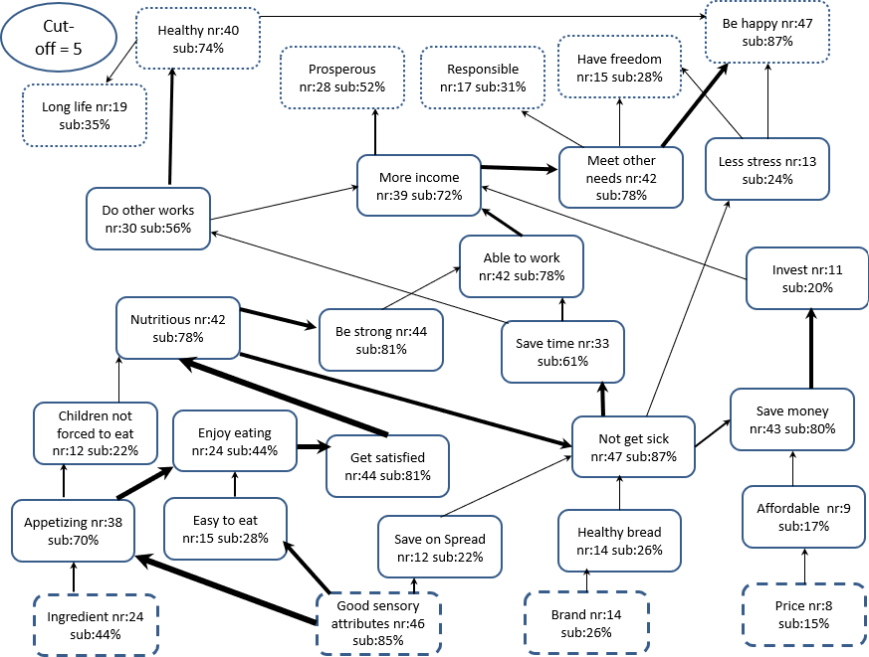
The hierarchical value map (HVM) of attributes, consequences and values associated with purchase of orange‐fleshed sweet potato bread (*N* = 51); nr and sub are, respectively, number and % of respondents identifying a construct; cut‐off = number of responses required to include a construct in HVM. The boxes represent: broken line = *attributes*, solid line = *consequences* and dotted line = *values*. Size of arrow denotes relative importance of the construct. [Colour figure can be viewed at wileyonlinelibrary.com]

Unlike the traditional neoclassical economic analysis, the MEC analysis constructs relate to mental representations, hence the need to use the laddering interview technique to systematically ‘extract’ them from the consumers' sub‐conscious mind. Figure 2 presents the aggregate hierarchical value map (HVM) of the whole sample, including male and female purchasers. The most important characteristics (i.e. *attributes*) of the OFSP bread driving purchase fall into two broad categories, namely good sensory features and general product features (i.e. ingredients, brand name and price). Among these, ‘good sensory features’ was identified by more than 85% of the respondents among the top three reasons driving the decision to purchase OFSP. The sensory attributes most mentioned (during the laddering probes) were sweet potato flavour/taste, sweet potato smell, mouthfeel, sweetness/sugary and colour. These features were perceived to better/higher for the OFSP bread compared to the other types of bread.

These findings corroborated those from pre‐survey FGDs, which indicated that consumers perceived OFSP bread as being sweet and having an attractive colour. However, it is possible that the completion of the JAR scale prior to the laddering interviews and the subsequent bread tasting might have led to the ‘goal‐priming’ of the respondents about the sensory attributes. Okello *et al*. (2018) indicated that discussion of product features prior to its evaluation influences the overall product assessment. Thus, it is possible that the high ranking of these features by a large proportion of respondents was related to the priming experience. This might also be said of the product features – brand, ingredients and price – which were discussed during the study's introduction.

The dominant association (depicted by the size of arrows) of the bread features and its benefits in the minds of respondents are twofold. First, those linking sensory features to bread being ‘appetising’, leading the consumer to ‘enjoy eating’, ‘get satisfied’, ‘be strong,’ ‘be able to work’ and ‘have more income’ to meet other needs. These consequences were ultimately mentally associated with ‘being happy’ or ‘having freedom in life’, which are the life goals (*values*). Notably, ‘more income’ was also mentally associated with ‘prosperous’, mentioned in the context of being wealthy. Second, the major mental *attributes‐consequences* association was an off‐shoot of the first and relates to those linking ‘good sensory attributes’ with ‘easy to eat’, ‘enjoy eating’ and ‘get satisfied’ as before. However, ‘get satisfied’ was mentally associated with nutrition (i.e. nutritious), ‘not get sick’, ‘save money’ (which would have been spent on medical care), ‘invest’ and ‘get more income’ to meet other needs as before, ultimately resulting in happiness and prosperity. The map further shows strong mental associations between ‘not get sick’ with ‘save time’, which is used to ‘work’ and ultimately being ‘healthy’ and ‘happy’.

Among the product features, ‘ingredients’ was ranked among the top three reasons for purchase of OFSP bread by 44% of the respondents. This attribute was mentioned in the context of the bread being rich in vitamin A and was mentally associated with health‐related benefits (*consequences*), namely ‘nutritious’ (78%), ‘not get sick’ (87%), ‘be healthy’ (74%). This finding showed that the respondents mentally associated the purchase and consumption of OFSP bread with health‐seeking behaviour. As predicted by the neoclassical economic theory, the price of OFSP also influenced the decision to purchase OFSP bread, but only among 15% of the respondents. For the latter sub‐group, the major concern was on ‘affordability’ and need to ‘save money’ which was mentally associated with ‘invest’(80%) and ultimately to attaining a life goal of being ‘prosperous’ (52%), ‘responsible’ (31%) and ‘having freedom’ (28%). The *value* ‘responsible’ was mentioned in the context of one’s capability to meet family obligations.

**Figure 3:**
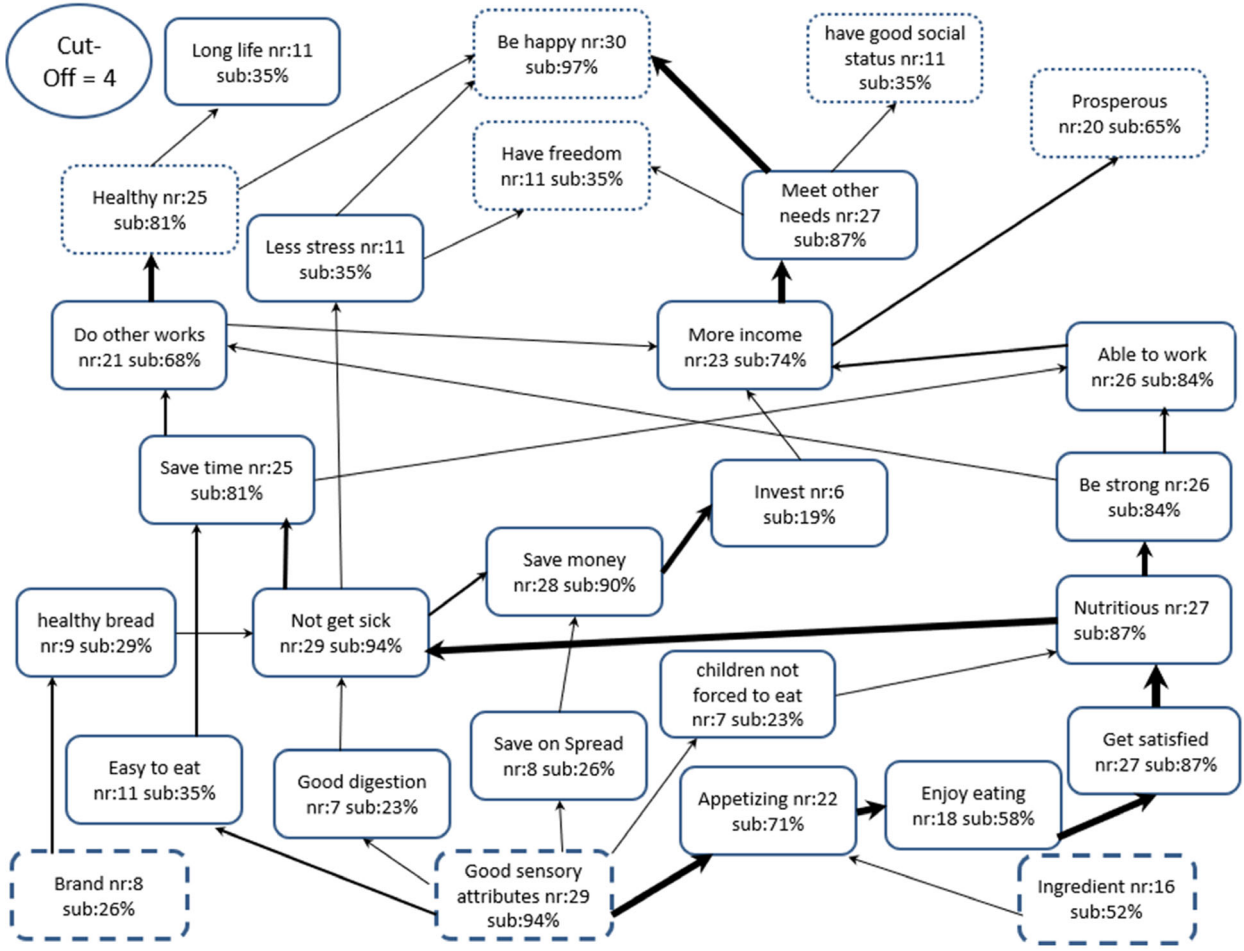
Female respondents’ hierarchical value map (HVM) of attributes, consequences and values associated with purchase of orange‐fleshed sweet potato bread (*n* = 31); nr and sub are, respectively, number and % of respondents identifying a construct; cut‐off = number of responses required to include a construct in HVM. The boxes represent broken line = *attributes*, solid line = *consequences* and dotted line = *values*. Size of arrow denotes relative importance of the construct.

**Figure 4:**
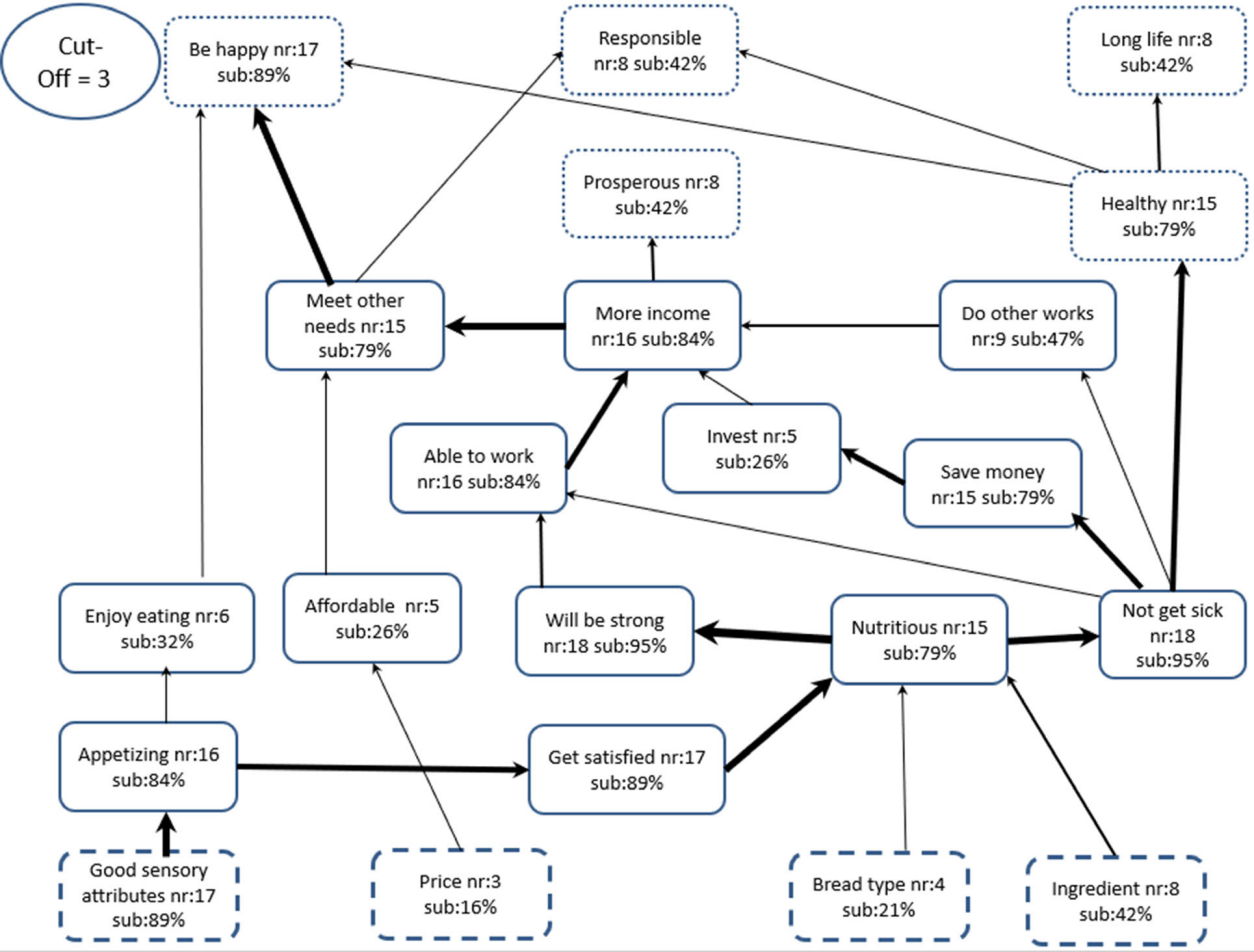
Male respondents’ hierarchical value map (HVM) of attributes, consequences and values associated with purchase of orange‐fleshed sweet potato bread (*N* = 20); nr and sub are, respectively, number and % of respondents identifying a construct; cut‐off = number of responses required to include a construct in HVM. The boxes represent: broken line = *attributes*, solid line = *consequences* and dotted line = *values*. Size of arrow denotes relative importance of the construct.

The HVMs for female and male respondents are presented in Figs 3 and 4, respectively. The maps show that a large majority of both female (94%) and male (89%) respondents were motivated to purchase OFSP bread by ‘good sensory attributes,’ as in the case of the aggregate HVM. The female respondents, however, mentally linked the decision to purchase OFSP bread with a new attribute, namely ‘bread type’. There were several other marked differences between male and female HVMs. First, the male HVM had fewer *consequences* (13) compared to the female HVM (17), suggesting greater complexity in the way mental constructs relating the purchase of OFSP bread are linked for female consumers than their male counterparts. Second, the female HVM captured a new *value*, ‘have good social status’, which was mentioned in the context of gaining respect and a good reputation in society. This *value* was mentally linked to the *consequence* ‘meet other needs’ and hence was associated with catering for family needs and, therefore, was seen to be a successful family person by society. Third, only the male HVM had the attribute ‘price’ which as before, was mentally linked to ‘affordable’, leaving some of the income to be spent on ‘other needs’, which was ultimately mentally associated with ‘being responsible’ and ‘being happy’. Clearly, there were differences in the psychosocial factors driving the decision to purchase OFSP bread between male and female purchasers. There were also significant differences by gender in the organisation of mental constructs relating to purchase of OFSP bread.

## Conclusions and implications

This study used the JAR, PA and MEC analyses to examine the quality and psychosocial factors influencing the purchase of bread made from OFSP, a biofortified product. The study was conducted among bread consumers recruited at the point of purchase in a major retail chain – Tuskys’ Supermarket – which was the sole producer and retailer of the bread at the time of the study. The OFSP bread, then marketed only in Nairobi, was in its infancy and a new concept to consumers, hence the choice of the analytical techniques.

The results of JAR and PA analyses showed that the acceptance, hence the decision to purchase OFSP bread, was affected by several sensory characteristics. Foremost among these were sweet potato smell, sweet potato flavour, hard texture and sourness, which were rated as being ‘too little’ by a significant percentage of both male and female consumers. The PA analysis further found that the consumers rated saltiness and sweetness/sugariness as being ‘too little’. The MEC analysis results corroborated the finding regarding the importance of sensory characteristics of OFSP bread, with more than 85% of the respondents listing them as major drivers of their purchase decisions, and only a few (17%) mentioning price. A smaller, but significant, proportion (40%) of consumers also considered ingredients. The sensory characteristics conferred several benefits (especially appetite, satisfaction, not‐getting‐sick, being strong hence able to work and making more money) to the consumers. These benefits were in turn associated with several life goals consumers aspire for, including prosperity, good health, good social status in the society, happiness and long life. This study, therefore, concluded that the purchase of OFSP bread was driven by its inherent quality characteristics, of which sensory attributes play a major part. It also concluded that consumers of OFSP bread are motivated by psychosocial factors, including the ultimate life goals/values they aspire for, and that price, a key economic factor in product purchase decisions, played a rather minor role among the existing bread purchasers.

Several implications have been drawn from the results of this study. First, results clearly provided a number of levers – in terms of quality characteristics – to pull in the refinement and reformulation of the OFSP bread. Addressing quality attributes rated as either ‘too little’ or ‘too much’ would further increase the demand and expand the market for the OFSP bread. Second, the finding that some of the health‐associated quality characteristics – saltiness and sugariness/sweetness – was rated as being on the lower side provides bakers and marketers with a tool for segmentation of the market by exploiting the niche market of the health‐seeking/conscious consumers. Moreover, advertisements explaining the benefits of low salt and sugar content in bread could increase consumer knowledge and potentially expand the client base. Third, the finding that sweet potato flavour and hard texture were ‘too little’ and wheat flavour ‘too much’ implies that there is still room to increase the proportion of OFSP purée in the bread. This would reduce reliance on more expensive wheat flour. Kenya is currently a wheat importer; hence, substituting more OFSP purée for wheat in bread making could reduce the wheat import bill. Fourth, the finding relating to life goals associated with the purchase of OFSP bread provides marketers with the tools for designing future of promotional/campaign messages for stimulating demand for the bread among the wider bread consuming population.

## Conflict of interest

The authors declare no conflict of interest in this work.

## Author contributions

**Julius Juma Okello:** Conceptualization (equal); data curation (lead); formal analysis (equal); investigation (supporting); methodology (equal); supervision (equal); writing‐original draft (lead); writing‐review & editing (equal). **Felix Mwala Shiundu:** Data curation (equal); investigation (lead); writing‐original draft (supporting). **Janet Mwende:** Data curation (equal); formal analysis (equal); investigation (supporting). **Carl Johan Lagerkvist:** Conceptualization (equal); formal analysis (equal); methodology (equal); writing‐review & editing (supporting). **Rose Adhiambo Nyikal:** Investigation (supporting); supervision (equal); writing‐review & editing (supporting). **Penina Muoki:** Conceptualization (equal); investigation (supporting); project administration (lead); supervision (equal); writing‐review & editing (supporting). **John Mburu:** Investigation (supporting); supervision (equal); writing‐review & editing (supporting). **Jan W. Low:** Writing‐review & editing (equal). **Guy Hareau:** Resources (equal). **Simon Heck:** Funding acquisition (supporting); project administration (supporting); resources (equal).

## Ethical approval

This study was conducted following the principles of the Helsinki Declaration on ethical non‐therapeutic research involving human subjects. All respondents provided written informed consent to participate in the study. The bread sample and water used in the study were approved for human consumption by the Kenya Bureau of Standards and were on display for sale to the consumers by Tuskys Supermarket at the time it was picked from the store shelf.

### Peer Review

The peer review history for this article is available at https://publons.com/publon/10.1111/ijfs.14822.

## Data Availability

Research data are not shared.

## Notes

^1^ Sweetpotato is a hexaploid crop – six pairs of chromosomes. Consequently, it is incredibly genetically diverse. Its flesh colour can be white, yellow, orange or purple. Orange‐fleshed sweetpotatoes, as the name indicates, are orange inside, indicative of a very high beta‐carotene content (typically 300–1200 retinol activity equivalent units per 100 g). The human body converts beta‐carotene into retinol or vitamin A, essential for good health. In the USA, only orange‐fleshed types are known. But in sub‐Saharan Africa, dominant varieties are white‐fleshed, with no beta‐carotene. OFSP is being bred through conventional means, combining beta‐carotene rich parents with local varieties with desired taste and agronomic (disease and drought resistance) traits.

^2^ Processed products with at least 15 μg g^−1^ product of trans‐β‐carotene were considered good sources of vitamin A.

^3^ Average exchange rate in 2018: 0.0099 US dollar for 1 KES. https://www.exchangerates.org.uk/KES‐USD‐spot‐exchange‐rates‐history‐2018.html.

^4^ The household head was defined as the person responsible for making most of the decisions concerning expenditure in the household.
